# 

**Published:** 1881-02

**Authors:** 


					﻿JVhole Number
CCCCXXXIV.
February, 1881.
Number 2,
Vol. XLII.
THE
CHICAGO
MEDICAL
T ournal and Examiner
EDITORS:
N. S. DAVIS, M.D., LL.D. JAS. NEVINS HYDE, A.M., M.D.
DANIEL R. BROWER, M.D.
CHICAGO:
PUBLISHED BY THE CHICAGO MEDICAL PRESS ASSOCIATION,
188 Clark Street.
Read the Notice of th'S Jot/'na' arror.r Advertisements.
				

